# Experimental investigation of size broadening of a K_α_ x-ray source produced by high intensity laser pulses

**DOI:** 10.1038/s41598-021-02585-5

**Published:** 2021-12-02

**Authors:** M. Gambari, R. Clady, L. Videau, O. Utéza, A. Ferré, M. Sentis

**Affiliations:** 1 LP3, CNRS, Aix Marseille Université, 13288 Marseille, France; 2 CEA, DAM, DIF, 91297 Arpajon, France; 3 Laboratoire Matière Conditions Extrêmes, CEA, Université Paris-Saclay, 91680 Bruyères-le-Châtel, France

**Keywords:** Lasers, LEDs and light sources, X-rays, Ultrafast lasers, Optics and photonics, High-field lasers, Characterization and analytical techniques, Nonlinear optics, Particle physics, Physics, Laser-produced plasmas

## Abstract

The size of a hard K_α_ x-ray source (${\mathrm{E}}_{{\rm{K}}_{\rm{\alpha }}}$ = 17.48 keV) produced by a high intensity femtosecond laser interacting with a solid molybdenum target is experimentally investigated for a wide range of laser intensity (I ~ 10^17^–2.8 × 10^19^ W/cm^2^) and for four values of the temporal contrast ratio (6.7 × 10^7^ < CR < 3.3 × 10^10^). Results point out the size enlargement of the x-ray source with the increase of laser intensity and with the deterioration of temporal contrast. It amounts up to sixteen times the laser spot size at the highest laser intensity and for the lowest temporal contrast ratio. Using hydrodynamic simulations, we evaluate the density scale length of the pre-plasma L/λ just before the main pulse peak. This allows us to show that a direct correlation with the laser absorption mechanisms is not relevant to explain the large size broadening. By varying the thickness of the molybdenum target down to 4 µm, the impact of hot electron scattering inside the solid is also proved irrelevant to explain the evolution of both the x-ray source size and the K_α_ photon number. We deduce that the most probable mechanism yielding to the broadening of the source size is linked to the creation of surface electromagnetic fields which confine the hot electrons at the solid surface. This assumption is supported by dedicated experiments where the evolution of the size enlargement of the x-ray source is carefully studied as a function of the laser focal spot size for the highest contrast ratio.

## Introduction

Laser-plasma hard x-ray sources are a suitable alternative to conventional accelerator-based x-ray sources of high brightness^1,2^ and for applications requiring a high x-ray flux and a micrometric size such as x-ray phase contrast imaging^3,4^. Their characteristics and operating capabilities have been improved for decades^5–8^ especially thanks to the continuous progress of femtosecond lasers in terms of intensity, spatial and temporal beam quality, repetition rate, reliability and compactness. In particular, such x-ray sources of ultrashort pulse duration driven by femtosecond laser systems offer an intrinsic synchronization for pump and probe experiments like time-resolved x-ray diffraction^9^ and time-resolved x-ray absorption spectroscopy^10^.

Laser plasma hard K_α_ x-ray sources are generated by the interaction of a high intensity laser pulse (I ≥ 10^16^ W/cm^2^) with most generally a high Z solid target. A spectrum composed of a large x-ray Bremsstrahlung emission dominated by a spectrally narrow K_α_ line is obtained coming from the interaction of hot electrons accelerated in the laser-induced plasma with inner shell electrons.

In first approximation, the laser plasma K_α_ x-ray source size S at full width at half maximum (FWHM) can be directly related to the size of the FWHM laser focal spot diameter d. Their ratio, S/d, approaches a value reported at best close to one^11^. This minimum ratio is generally obtained for a rather low laser intensity on target and a high value of the laser temporal contrast ratio (CR). The latter is here classically defined as the main pulse peak intensity (fs) over the background Amplified Spontaneous Emission (ASE, ns) intensity, such as CR = I_peak_/I_ASE_. Many studies have shown that the source size ratio S/d increases with the laser intensity^4,12–14^. The smallest K_α_ x-ray source size produced by laser plasma reported today is ~ 6 µm with a ratio × 4.6, using a massive silicon target and a p-polarized laser beam of 30 fs duration focused with a nominal incidence angle of 45° and with an intensity up to 5 × 10^17^ W/cm^2^ at λ = 800 nm^15^. By applying cleaning pedestal techniques or second harmonic conversion of the laser, studies have shown that an improvement of the contrast ratio leads to a pronounced decrease of the x-ray source size^13,16–18^. However, at shorter laser wavelength (λ ~ 400 nm), the reduced kinetic energy acquired by the hot electrons is less favorable for x-ray generation since the laser field ponderomotive potential scaled as Iλ^2^ ^19^. Among these works only very rare studies have been published concerning the impact of different values of the temporal contrast ratio, at the fundamental wavelength of 800 nm, on the evolution of the x-ray source size for a large laser intensity range^13^.

The mechanisms of x-ray source size enlargement compared to the focal spot size in relation with the driving laser parameters are still not well understood. These mechanisms rely on the transport and energy of the hot electrons which are very complex to study both experimentally and numerically. The principal explanations provided in the literature are the divergence angle of propagation of hot electrons depending mainly on the laser intensity and the laser incidence angle with the target surface, and their larger stopping path inside the target when the laser intensity increases^20,21^. Other formulated hypotheses argue the wings of the laser focal spot^21,22^, and/or the surface electromagnetic fields effects^23,24^ which induce a lateral propagation of hot electrons along the target surface^25,26^. Besides, space-charge inhibition of hot-electron transport can reduce dramatically their penetration in the target^27,28^. These collective effects become increasingly important for laser intensities I > 10^18^ W/cm^2^ ^29^. It is therefore instructive to study the penetration depth of hot electrons and their trajectories (divergence angle) inside the solid. Hot electron energy and their angular distribution can be measured by imaging electron energy-sensitive plate stacks or an electron spectrometer. They can also be measured by indirect techniques such as coherent transition radiation^30^ at the rear target surface when using thin foils, or K_α_ fluorescence measurement from multi-layer targets composed of materials of different atomic numbers Z^31–33^. However, the effect of varying the thickness of a single Z foil target on the x-ray source size and K_α_ photon number has hardly ever been reported.

The main objective of the present work is to bring original experimental measurements of the size of a K_α_ x-ray source as a function of the two main laser driving parameters, e.g. the laser intensity and its temporal contrast ratio. Compared to the literature, we intend to go one step further by expanding such parametric study to a wide number of temporal contrast values (4 CR, from 6.7 × 10^7^ < CR < 3.3 × 10^10^) and a larger range of laser intensity (I ~ 10^17^–2.8 × 10^19^ W/cm^2^) with the same experimental setup, and also by providing dedicated experiments to progress in knowledge and understanding of the characteristics of a K_α_ x-ray source driven by high intensity ultrashort lasers. So, the correlation between the x-ray source size and the hot electron path inside the solid target is investigated by varying the thickness of a molybdenum foil as target down to 4 µm and the laser focal spot size is varied for two fixed laser energies and one fixed laser intensity to study its impact on the x-ray source size for the best CR. Crossed analysis of obtained results with the help of literature confirm the prevalence of surface electromagnetic fields among others mechanisms for explaining the large size broadening of the x-ray source generated at high laser driving intensity even for temporal contrast as high of 3.3 × 10^10^. Finally, this large and detailed experimental study of the size broadening of the x-ray source as a function of laser driving characteristics can be used for theoretical future studies and simulations, especially to gain insight into electron transport and for the perspective of optimizing the brilliance of such hard x-ray source induced by laser-driven plasmas at very high laser intensity.

### Experimental setup

The 10 Hz—20 TW—800 nm laser beamline of ASUR facility is used for the study^34^. The p-polarized, $\sim$25 fs laser pulse is focused at an incidence angle of 45° with a f/4.5 off-axis parabolic mirror on a molybdenum (Mo, Z = 42, E_Kα_ = 17.48 keV) solid disk or foils of 100 mm diameter and of variable thickness. Note that a fresh target surface is provided for each laser shot. At 45° incidence angle, laser absorption through vacuum heating mechanism is optimized when the pre-plasma density scale length L is small compared to the laser wavelength λ^35^. The laser focal spot diameter is d_x_ × d_y_ = 4.9 × 4.6 µm^2^ at FWHM. The energy is varied from ~ 1 mJ up to ~ 254 mJ on target with ~ 40% of the encircled energy at FWHM. By considering the real 2D beam profile, laser peak intensity on target surface vary from ~ 10^17^ W/cm^2^ up to ~ 2.8 × 10^19^ W/cm^2^. The temporal contrast ratio CR is modified by using up to three saturable absorbers. It varies from 6.7 × 10^7^ to 3.3 × 10^10^ for a measured ASE level at t = −480 ps before the peak of the main pulse (see “Methods”, Fig. 6).

The FWHM effective size of the x-ray source is determined by the knife edge technique assuming a gaussian shape^12^. Due to the geometrical configuration of the targetry system which ensures a fresh surface for each laser shot^12^, all the defects related to the geometric imperfections of the target and of its displacement system induce a more pronounced widening of the x-ray effective source size in the horizontal dimension compared to the vertical one. This is particularly significant when multipulse laser irradiation (~ 20 to ~ 1000 laser shots) is required to acquire an image of the knife edge. Thus, we only measure the vertical diameter of the x-ray source since the objective is to link the enlargement of the x-ray source size with physics phenomena and not to mechanical displacement and/or geometrical surface defects. The target characteristics are reported in “Methods”. Finally, the absolute number of K_α_ photons per pulse is deduced from reconstructed x-ray spectrum obtained using the photon counting method^36^ in which a x-ray CCD camera (PIXIS-XB from Princeton Instruments, 1024 × 1024 pixels of 13 × 13 µm^2^ pixel size) acts as a dispersive spectrometer.

## Results and discussion

### Impact of the temporal contrast and laser intensity

**Figure 1:**
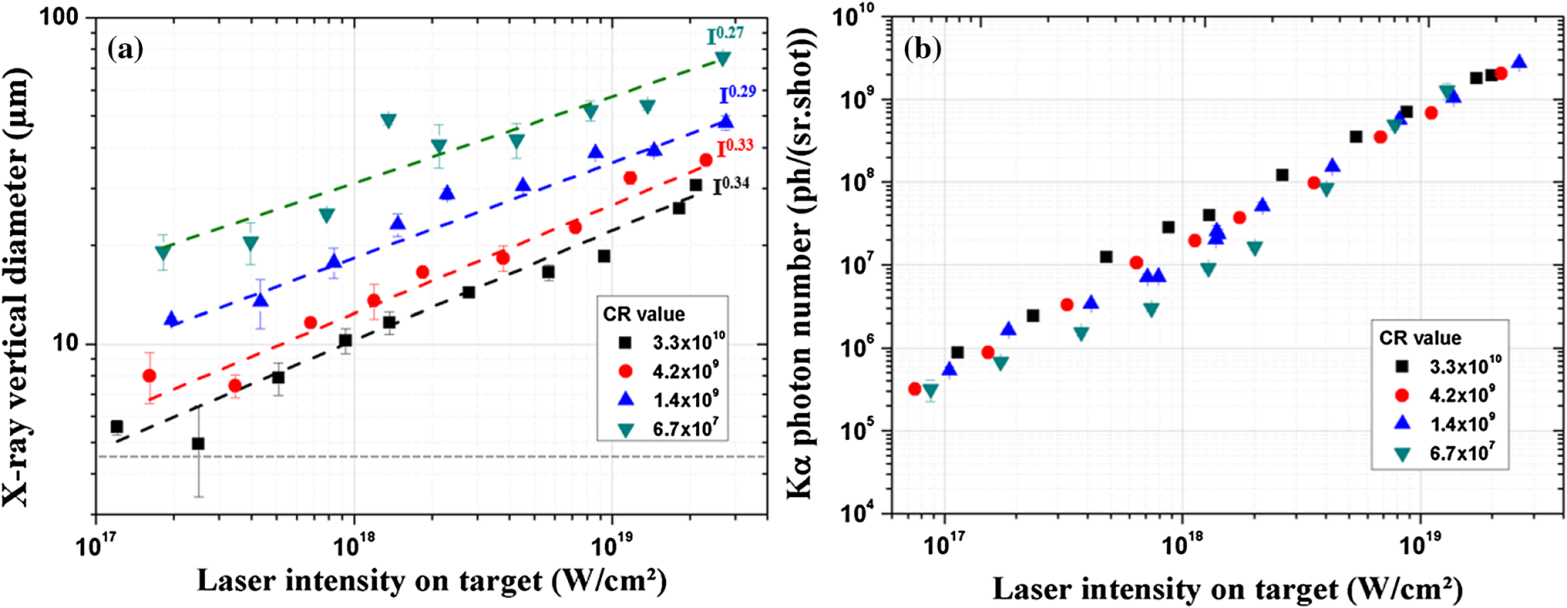
**(a)** FWHM x-ray vertical diameter versus laser intensity on target, for four temporal contrast ratio values. The error bars correspond to the standard deviation of the average FWHM line spread function measured for three different zones of the knife edge image. The dashed lines for the x-ray source diameter data correspond to a I^ε^ power fit with ε varying with the contrast ratio value. The grey horizontal dashed line represents the FWHM vertical laser focal diameter (~ 4.6 µm). **(b)** K_α_ photon number versus laser intensity on target, for four temporal contrast ratio values. The error bars are given by the standard deviation calculated from three independent measurements of N_K__α_.

The vertical size of the x-ray source and the K_α_ photon number per steradian and per shot, N_Kα_, are reported in Fig. 1a,b respectively, for four CR values (3.3 × 10^10^, 4.2 × 10^9^, 1.4 × 10^9^, and 6.7 × 10^7^), and as a function of laser peak intensity. The peak intensity is varied by changing the laser pulse energy while the duration and the laser spot size on target are kept unchanged.

In Fig. 1a, we first observe that the evolution of the x-ray source size, S, as a function of the laser intensity on target can be fitted using a power law with S $\propto$ I^ε^. This behavior has already been put forward in our previous works^4,12^, but only for one temporal contrast ratio value (CR ~ 10^10^). Here, we show that it can be extended to all the temporal contrast values explored in the present study and that the ε factor decreases from 0.34 to 0.27 when CR is deteriorated. We also observe that the higher is the temporal contrast value the smaller is the source size for the whole laser intensity range. Secondly, at relatively low laser intensity (~ 10^17^ W/cm^2^ to 3.0 × 10^17^ W/cm^2^), the smallest x-ray source size, S ~ 5 µm, is obtained for the highest temporal contrast ratio (3.3 × 10^10^) corresponding to only × 1.1 the laser focal spot size. It is the smallest Mo-based K_α_ laser plasma x-ray source size reported up to now at the fundamental Ti:Sa wavelength (λ = 800 nm). This is due to the high contrast of the laser driver in addition to the large numerical aperture of the focusing parabola. Inversely, for the lowest CR (6.7 × 10^7^), the x-ray source size reaches 18 µm. This spatial enlargement is correlated to the degradation of the contrast ratio since the measurement of the laser spatial profile for the four CR show no noticeable difference (see “Methods”, Fig. 7). When the laser intensity increases, the source size increases up to ~ 76 µm for I = 2.8 × 10^19^ W/cm^2^ and the lowest CR. However, at very high laser intensities, the difference between the source sizes tends to reduce for the different temporal contrasts, as shown by the decrease of the parameter ε for decreasing ASE contrast value (Fig. 1a). Indeed, for I < 3.0 × 10^17^ W/cm^2^, S_CR = 6.7×10_^7^/S_CR = 3.3×10_^10^ ~ 4, while this ratio is equal to ~ 2.5 for I > 1.0 × 10^19^ W/cm^2^.

Conversely, in Fig. 1b, we observe that the number of produced K_α_ photons, N_K__α_, is independent of the CR value for lower (I ≤ 1.0 × 10^17^ W/cm^2^) and higher (I > 3.0 × 10^18^ W/cm^2^) laser intensities, and slightly CR-dependent at intermediate laser intensities where we notice a difference with a lower K_α_ photon number produced when CR deteriorates. In a previous work we already observed a similar behavior and explained it by the competition and transition between different laser absorption mechanisms governing the generation of hot electrons like resonant absorption, vacuum heating or J × B heating^34^. Their dominance depends on the pre-plasma density scale length L induced by the ASE and pedestal at the front of the solid target and on the laser intensity^35,37^.

**Figure 2:**
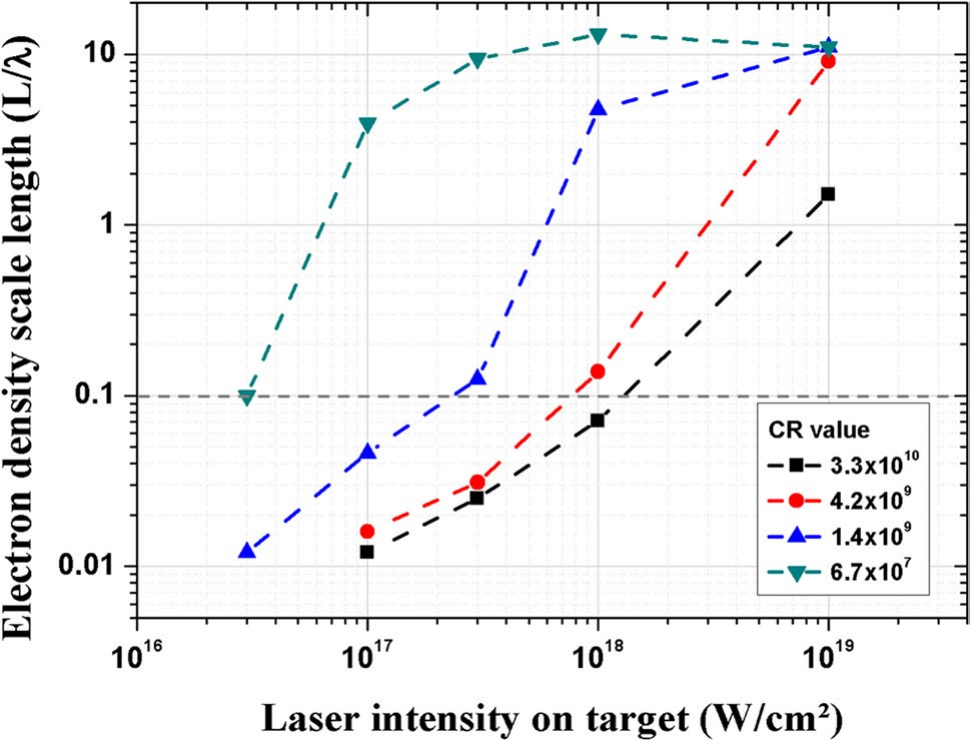
Simulated pre-plasma density scale length L normalized by λ for the four experimental CR studied and from I = 3.0 $\times$ 10^16^ W/cm^2^ to 1.0 $\times$ 10^19^ W/cm^2^. At relativistic laser intensity regime (I > 2.2 × 10^18^ W/cm^2^), the Lorentz factor γ must be considered in the calculation of the critical density n_c_, giving a measured L at I = 10^19^ W/cm^2^ lower than the one at I = 10^18^ W/cm^2^ for CR = 6.7 × 10^7^. The grey horizontal dashed line represents L/λ = 0.1.

In support of the experiment, the low fluence Lagrangian one-dimensional hydrodynamic code ESTHER^38^ is used to simulate the plasma induced by both ASE and laser rising edge up to 1.2 ps before the peak intensity. The ESTHER code is currently used for such applications in this intensity regime^39–41^. This allows us to investigate the influence of the pre-plasma length, L, and therefore of the laser absorption mechanisms on the x-ray source size and the K_α_ photon number. The simulation considers the temporal evolution of the laser intensity as measured with a third order autocorrelator (see “Methods”). L is defined using the simulated electron density profile n_e_ normalized by the critical density n_c_ (~ 1.72 × 10^21^ cm^−3^ at λ = 800 nm) as a function of laser intensity, from 3.0 × 10^16^ W/cm^2^ to 1.0 × 10^19^ W/cm^2^ and for the four temporal contrast ratios experimentally studied. It corresponds to a range of maximum ASE and pedestal intensities of ~ 3.0 × 10^11^ W/cm^2^ to 1.0 × 10^14^ W/cm^2^ (see “Methods”, Fig. 6). An example of electron density profile is given in Fig. 8 in “Methods”. Figure 2 gives the different values of L/λ at −1.2 ps before the laser intensity peak for the four experimental temporal contrasts and five laser intensities.

We observe that the density scale length L/λ varies almost by three decades from ~ 0.02 at the lowest intensities and CR ≥ 1.4 × 10^9^ up to 10 at the highest intensities and CR < 3.3 × 10^10^. As described by Gibbon et al*.*^35,42^, we deduce in a first approximation that at low laser intensities, the dominant absorption mechanism for at least 3 CR values is vacuum heating (L/λ < < 0.1) which progressively changes to resonant absorption (L/λ > 1) for higher intensities^35^. However, the light pressure P_L_, which cannot be included in the ESTHER simulations, becomes to be significant at high laser intensity and has to be taken in consideration. Indeed, it progressively crushes the plasma, thus providing conditions (L/λ < < 0.1) for which the absorption of the laser energy through vacuum heating and J × B heating becomes highly dominant^35,37^. The laser intensity I_0,_ for which P_L_ = P_th_, can be analytically estimated as I_0_ = $\frac{{\rho {\rm c}}}{2{{\rm cos}\theta }}$(9.45 × 10^3^)^2^ ${\mathrm{I}}_{\rm{ASE}}^{1/2}$ (see “Methods”). When the laser intensity I exceeds I_0_, the radiative pressure pushes the pre-plasma back to the target, resulting in a steepening of the electronic density gradient and consequently the mechanisms of laser absorption are modified^43,44^. We evaluate this compression at ~ 500 fs before the time corresponding to the maximum peak intensity (see “Methods”, Fig. 6). For I > 3 × 10^18^ W/cm^2^ and for all CR, the calculation gives L_d_/λ < < 0.1.

In brief, for all CR the same absorption mechanisms are dominant at least for the lowest and highest laser intensities. This analysis explains why the number of K_α_ photons observed on Fig. 1b is independent of CR at low and high intensities since heating electron mechanisms are the same, as demonstrated. However, the x-ray source size which depends on the trajectories of hot electrons strongly varies with CR values (see Fig. 1a) even for the same laser intensity and as shown for the same absorption mechanism. This preliminary conclusion motivates further dedicated experiments presented hereafter in order to investigate such spatial broadening phenomena of the x-ray source.

### Impact of the laser beam spatial contrast

**Figure 3:**
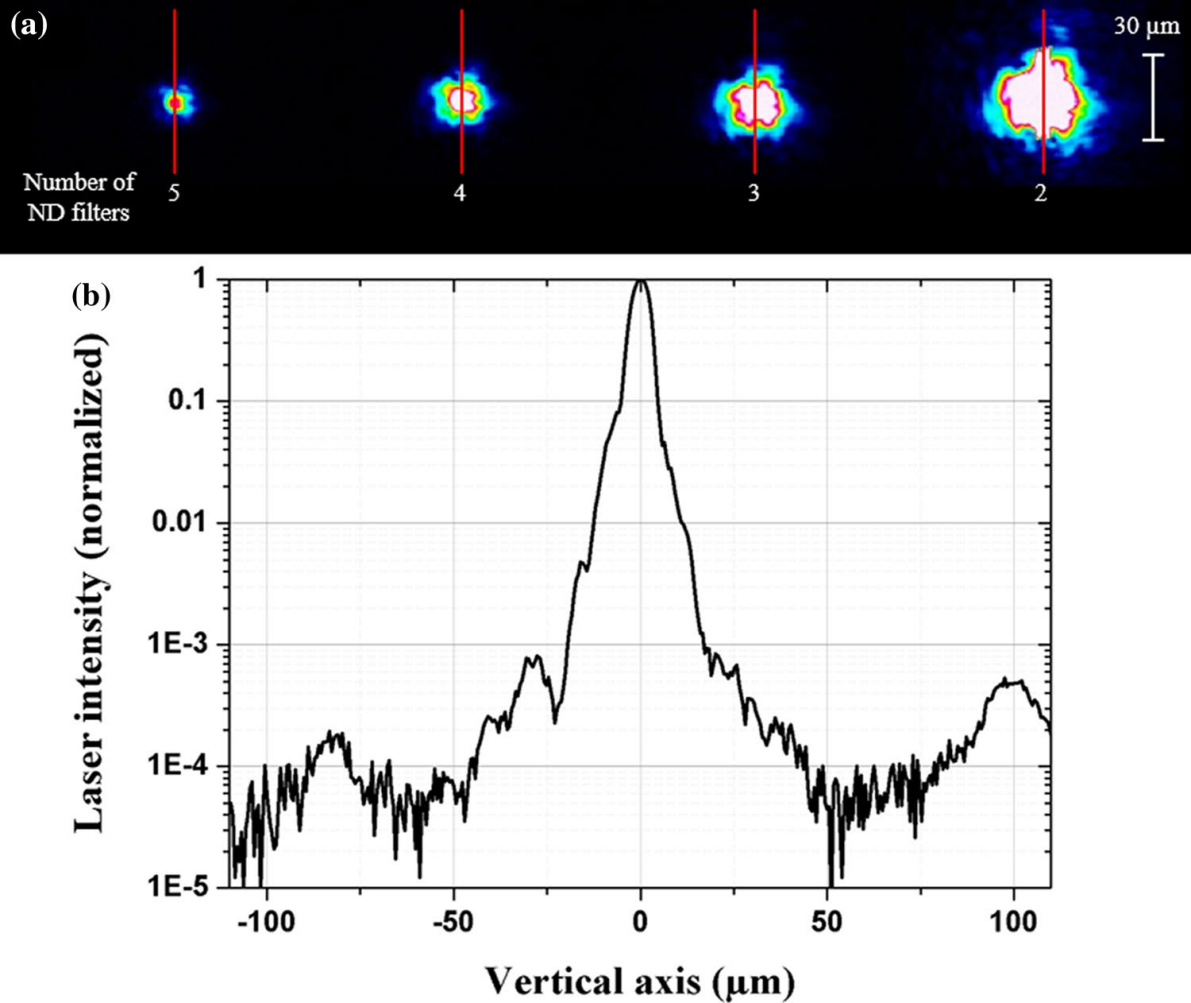
**(a)** Snapshots of the laser spatial beam distribution. Differences between each snapshot correspond to the removal of one neutral density filter. **(b)** Stacked vertical laser focal spot size profile coming from the red line profile on the images presented in **(a)**. Wings are present and their intensities are I < 5.0 × 10^16^ W/cm^2^, even when the laser peak intensity is measured at I ~ 1.0 × 10^19^ W/cm^2^.

In this section, the increase of the x-ray source size is studied in correlation with the growing importance of the laser beam wing size at very high laser intensity. For this purpose, the spatial distribution of the focal spot intensity is characterized on a large dynamic range (> 10^4^) by removing calibrated neutral density filters in front of the beam analyzer detector (see “Methods”), as shown in Fig. 3. Note that the transmission of a single neutral density filter is ~ 16.7% at λ = 800 nm.

As show in Fig. 3, low intensity wings are spread over 100 µm from the laser focal spot center. In fact, for a very high laser peak intensity I = 1.0 × 10^19^ W/cm^2^, the intensity of these wings is inferior to 5.0 × 10^16^ W/cm^2^. If we make the simplified assumption of the locality of the x-ray emission in concordance of the local laser intensity value the K_α_ photon number produced in the zone excited by the wings of the laser is at least ~ 10^4^ lower than in the zone corresponding to the laser peak intensity (I = 1.0 × 10^19^ W/cm^2^) as we can extrapolate from Fig. 1b. Therefore, these photons are not even experimentally detected, and we consider that the contribution of these wings in the x-ray emission can be neglected even at very high laser intensity. These observations rule out this hypothesis based on the spatial beam contrast which was discussed in literature^21,22^ to explain the enlargement of the x-ray source in our operating conditions.

### Impact of the foil target thickness

Hot electron scattering in a solid is dependent on their temperature T_h_ and their energy E_h_ and therefore on the laser intensity^35,45,46^. Since the generated hot electron beam presents a divergence angle, an enlargement of the x-ray source size is thus expected at high laser intensities due to their larger penetration depth^46–48^. As a result, experiments where the target thickness is varied would serve to investigate the effects of the hot electron trajectories inside the target on the x-ray source size broadening. More precisely, it would be expected that reducing the thickness of the target to micrometric values should lead to a reduction in the x-ray source size and of the number of K_α_ photons produced in comparison with a massive target, at least for high laser intensities.

**Figure 4:**
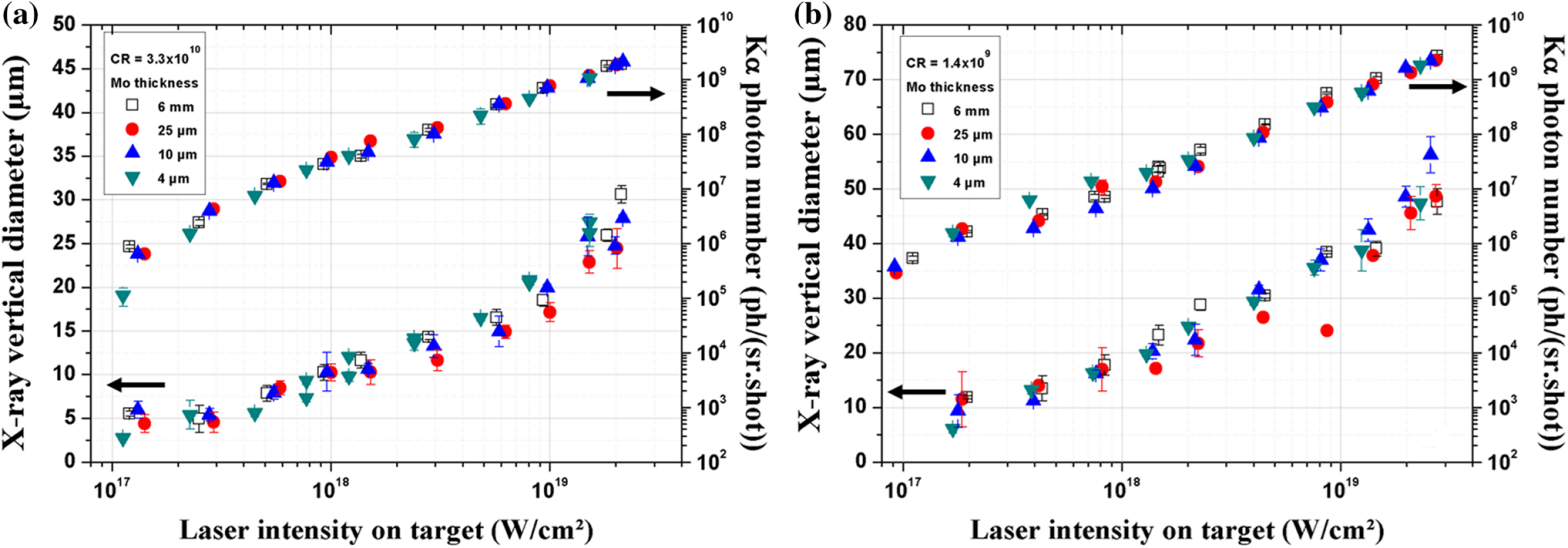
Comparison of the x-ray vertical source size and the Kα photon number versus laser intensity between a 6 mm massive Mo target and Mo foil targets of 25 µm, 10 µm and 4 µm thickness. For CR = 3.3 $\times$ 10^10^ in (**a**) and for CR = 1.4 × 10^9^ in (**b**).

In order to choose the thickness of the target foil, the depth for which a produced K_α_ photon will not escape from the Mo target due to its absorption in the solid is determined. Practically, one estimates the depth for which the K_α_ photon number is divided by the factor e. Using the mass attenuation coefficient for a Mo K_α_ photon propagating in a Mo target (µ/ρ ≅ 20 cm^2^/g yielding to µ ≅ 203.4 cm^−1^)^49^, the absorption length in Mo is ≅ 50 µm. The x-ray source size and the K_α_ photon number are consequently studied for three molybdenum target thicknesses below this length (25 µm, 10 µm and 4 µm) where re-absorption phenomena do not significantly occur, and compared with the previous results obtained with the 6 mm target presented in Fig. 1. Results are reported in Fig. 4 for two contrast ratio values of 3.3 × 10^10^ and 1.4 × 10^9^.

In Fig. 4a, we notice that the x-ray source size and N_K__α_ evolution are independent of the target thickness for both CR. For the x-ray source size, a maximum variation of × 1.2 between the different target thicknesses is observed in most cases. Thus, these experimental results suggest that the hot electron penetration depth is less than 4 µm. Note also that for the foil targets, we further checked if these results could not be explained by the refluxing effect at the target rear surface^8^. For inhibiting this effect, we set a SiO_2_ substrate placed behind and in close contact with the Mo foils. Results of the evolution of S and N_Kα_ are again identical with and without the substrate ruling out this hypothesis.

The electron penetration range is generally limited both by collisional and collective effects. Collisional stopping power is related to the electron beam collisions with the propagation medium material while the collective effects come from the existence of self-generated return currents provided by the hot electrons^31,50^. Based on Bell et al*.* studies^24^, Volpe et al*.* have shown for an aluminum target that the collective effects start to compete with collisional effects for laser intensities ~ 10^17^ W/cm^2^ and become dominant, over collisional processes with the ions and electrons of the target medium, for intensities ~ 10^18^ W/cm^2^ ^28^. Here we use the analytical description proposed by Volpe et al*.* to calculate the total electron penetration depth, R_total_, taking into account collisional and collective effects from the harmonic mean, ${\mathrm{R}}_{\rm{total}}= \frac{{\rm{R}}_{\rm{collisional}}{\mathrm{R}}_{\rm{collective}}}{{\mathrm{R}}_{\rm{collisional}}+{\mathrm{R}}_{\rm{collective}}}$ (see “Methods”). For I = 1.0 × 10^17^ W/cm^2^ and 2.0 × 10^19^ W/cm^2^, the maximum electron penetration depth R_total_ is 0.13 µm and 2 µm respectively, while R_collisional_ would be ~ 0.35 µm and 7.6 µm without considering the collective effects. R_total_ is lower than the thinnest target thickness used in the present experiment (4 µm). This electron penetration depth limitation induced by collective effects may therefore explain why in our experimental condition N_Kα_ is independent of the target thickness. However, this phenomenon cannot explain the observed enlargement of the x-ray source size.

Reich et al*.* have observed similar experimental results, consisting of a large broadening of the K_α_ source size compared to the laser focal spot size. Their explanation is the existence of strong self-induced fields which redirect the hot electrons over the target surface^21^. Later for experimental conditions very similar to ours, Li et al*.* observed fast electrons emitted along the surface (SFE) with a relatively large ratio between the number of SFE to the total number of hot electrons (17% to 28%)^51^. Their 2D-PIC numerical simulations reveal that they are induced by surface quasistatic electromagnetic fields which confine them at the target surface. In addition, it has been shown that these SFE are dependent of laser intensity and temporal contrast^52^. In view of these observations and of the analyses of the evolution of the x-ray source size as a function of the target thickness, we thus suggest that the trajectory of hot electrons propagating through the solid target might be bent by magnetic fields in the interaction region and the overdense plasma.

### Impact of the laser focal spot size

**Figure 5:**
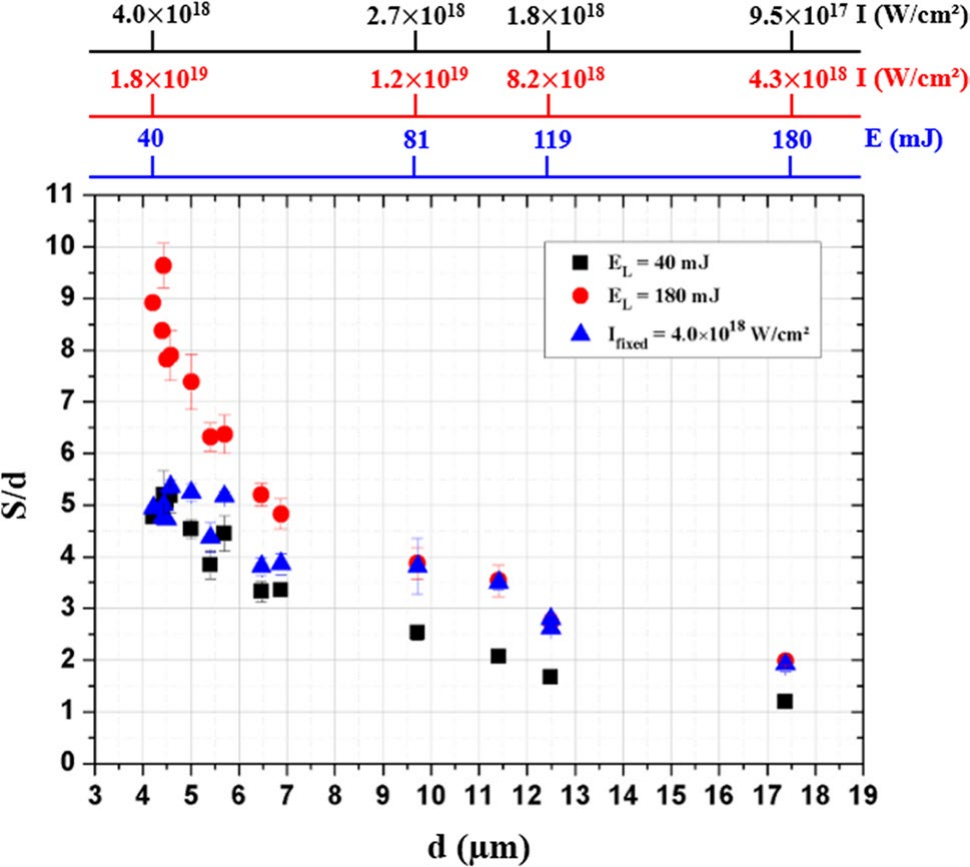
Ratio between the x-ray source size (S) and the laser focal spot size (d) versus laser focal spot size for two constant laser energies (40 mJ—black square and 180 mJ—red point) and for a fixed maximum laser intensity on target (4.0 × 10^18^ W/cm^2^—blue triangle).

The divergence of the hot electron beam formed by the laser-induced plasma is in part due to the variation of the laser intensity along the laser focal spot profile^53^. Considering also that the surface magnetic field is dependent on the focal spot size^54^, it is instructive to evaluate the influence of the laser focal spot size on the x-ray source size. In this perspective, the laser spot size is varied by moving the parabola along the focal axis toward the target or away from it, corresponding to a defocus range of ± 100 µm. This is superior to the experimental Rayleigh length z_R_ = 67 µm when the laser beam is approximated as gaussian. However, when the beam is not at the best focus (in intermediate field), its spatial profile can no longer be considered as gaussian. Nevertheless, the beam diameter can still be measured, and the laser spot diameter is varied between ~ 5 and ~ 17 µm in our experimental conditions. This experiment is performed for the highest temporal contrast ratio (CR = 3.3 × 10^10^) and for three laser conditions: two laser energies fixed at E_L_ = 40 mJ and E_L_ = 180 mJ respectively, and a constant maximum peak intensity set at I_fixed_ = 4.0 × 10^18^ W/cm^2^ by compensating the increase of the focal spot size with the increase of the laser energy, considering the real 2D laser beam profile. Figure 5 shows the evolution of the ratio between the size of the x-ray source S and the size of the laser focal spot d as a function of d.

In Fig. 5, we show that S/d increases by a factor of ~ 2 when the laser energy increases by 4.5 whatever the size of the laser spot. Moreover, the smaller the focal spot size the higher is S/d, and finally when the focal spot size increases, S/d tends towards one. These experimental observations are in accordance with a scenario where the self-induced electromagnetic fields increase with laser intensity and are higher with tighter focal spots^54^. These fields will have the tendency to confine the hot electrons within the target along the surface as discussed above and thus to increase the size of the x-ray source.

Finally, our result presented in Fig. 5 may explain the low ratio S = 1.7d with larger laser spot size d = 15 µm, obtained by Fourmaux et al*.* for similar laser intensity (I = 6.0 × 10^18^ W/cm^2^)^15^. In this work, it was only attributed to the improvement of the CR. However, thanks to the Fig. 5, we show that low S/d ratios found in the literature can also be explained by the laser focal spot size itself with reduced effects of self-induced electromagnetic fields on hot electron trajectories for larger laser focal spots compared to smaller ones.

## Conclusion

In this paper, the x-ray source size enlargement is experimentally studied by varying the laser intensity on target, the temporal contrast of the laser pulse, the target thickness and the laser focal spot size. We observe that the x-ray source size is increasing with the laser intensity on target and the degradation of the temporal contrast. Since the ASE and pedestal intensity in a time scale of ns to ps is crucial in tailoring the plasma properties and electron heating, a 1D numerical simulation (ESTHER) is used to estimate the electron density scale length L/λ as a function of laser intensity and CR. At non-relativistic laser intensity regime, L/λ is extremely small (L/λ < < 0.1) except for the lowest CR = 6.7 × 10^7^. As the laser intensity increases, the laser pressure radiation limits the expansion of the electron density scale length and even leads to a gradient steepening in such a way that L/λ becomes very small at high laser peak intensity for all CR. As already demonstrated in previous works, the parameter L/λ is of prime importance in high intensity laser-plasma interaction because it allows to explain with confidence and in relation with the different laser absorption mechanisms the evolution of the number of K_α_ photons produced as a function of laser intensity and temporal contrast ratio. However, this parameter is not relevant to unambiguously describe the evolution of the x-ray source size because the transport of hot electrons also depends for a fixed laser intensity on collisions, collective effects and self-generated electromagnetic fields. In an original study, where the thickness of the Mo target is varied down to 4 µm and for a large range of laser intensity (~ 10^17^ W/cm^2^ to 2.0 × 10^19^ W/cm^2^), we show that both the evolution of N_Kα_ and the x-ray source size S are outright independent of the target thickness (from 6 mm down to 4 µm), whatever the temporal contrast studied. The limited penetration depth (< 4 µm) of hot electrons experimentally observed may be explained by considering the collective effects of the hot electrons scattered inside the solid target. In a last experiment, the laser focal spot size d is varied to study its influence on the x-ray source size enlargement. The reduction of the laser focus spot size increases the ratio between the size of the x-ray source and the size of the laser focus spot. The cross-interpretation of all our experimental results suggests that the enlargement of the size of the x-ray source is strongly linked to the presence of self-generated electromagnetic fields which tend to bent a significant part of the hot electrons generated by J × B heating or vacuum heating along the surface of the target.

Considering the optimization of the peak brilliance of such K_α_ x-ray source in relation with source size minimization, we show that trying to limit the x-ray source size enlargement by using target foils as thin as 4 µm does not bring any improvement in such experimental conditions even with a high temporal contrast. Working at high laser intensity allows to increase dramatically the number of produced x-ray photons. Additionally, working with a very high temporal and spatial contrast of the laser beam allows to limit the x-ray source size broadening. The transport of the hot electron to a much larger zone than the laser focal aera induced by the self-electromagnetic fields is intrinsic of such laser interaction with a solid target but this is limited with high temporal contrast ratio. The improvement of temporal contrast ratio to higher value than ~ 3.0 × 10^10^ by various techniques^55,56^ as already demonstrated may be relevant to still enhance the peak brilliance of the x-ray source.

Moreover, this wide set of experimental data on the evolution of the x-ray source size as a function of a large range of parameters (temporal contrast ratio, laser intensity, target thickness and laser focus beam size) with the same experimental setup can be useful for the development and validation of numerical studies. This includes a combination of PIC and Monte-Carlo approaches, which are far out of the scope of the present paper, to study in details the energy distribution and trajectories of hot electrons which remains still today not well understood. Indeed, when using such tightly laser focus and a laser pulse including ASE and pre-pulses at different time scales from ns to fs, 2D to 3D simulations are required. As an example, such simulation studies will be useful to try to explain the dependence of the x-ray source size to the temporal contrast ratio, experimentally shown as S ∝ I^ε^, where ε varies with the temporal contrast ratio.

## Methods

### Targets

The 6 mm massive Mo target thickness comes from *Kurt J. Lesker *Company. Its characteristics are: 99.95% purity, rugosity < 1 µm, and a flatness of ± 20 µm overall the 100 mm disk diameter. The 25 µm, 10 µm and 4 µm Mo targets come from *GoodFellow* with thickness variation of ± 25% below 10 µm, and ± 15% for 10 µm and 25 µm thickness. The molybdenum foils are held only in their center and on a few millimeters in the periphery of the outer diameter of the disk by a rotating support partially hollowed out. This latter also ensures the obtention of vacuum at the rear side of the targets. The deviation to a perfect flatness of the thinnest target (4 µm) is measured to be < 40 µm, which is lower than the Rayleigh range of the laser (67 µm), ensuring approximatively the same laser intensity on target between two consecutive shots. More details about the target positioning can be found elsewhere^12^.

### Measurement and modification of the temporal contrast ratio

**Figure 6:**
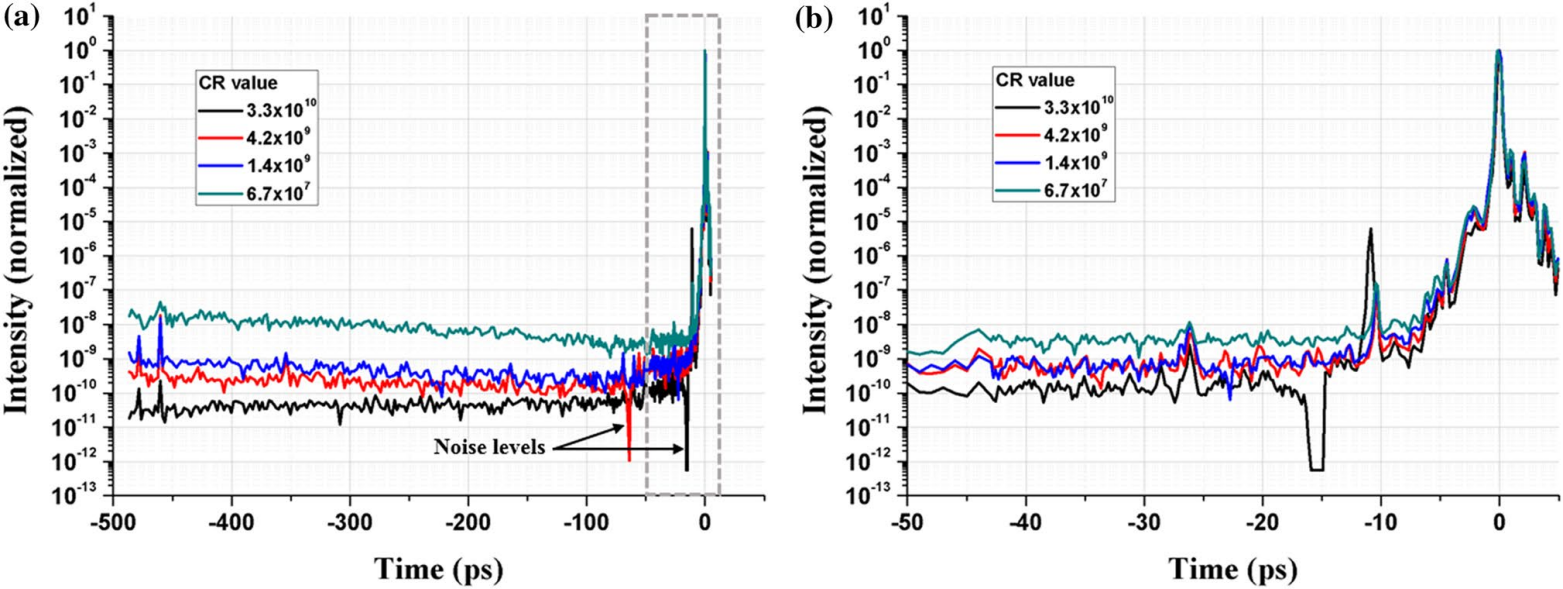
Temporal characterization of the laser pulses interacting with the Mo target. The main pulse corresponds to the time t = 0 ps and the ASE measurement goes up to −480 ps before the main pulse as shown in **(a)**. The CR is defined by I_peak_/I_ASE_. **(b)** Zoom up to −50 ps before the main pulse, corresponding to the grey dashed rectangular line in **(a)**.

The ASE and pre-pulses are measured up to 480 ps before the main pulse by a 3rd order autocorrelator (Sequoia, from *Amplitude Technologies*, see Fig. 6). Note that three pre-pulses are also present few ns ahead of the main pulse. However, they do not influence the x-ray generation as their contrast value is higher than 10^8^ ^34^. A pre-pulse around −12 ps is a measurement artefact coming from optics inside the Sequoia. The ASE duration is also measured up to 3 ns ahead of the main pulse thanks to the implementation of a ~ 1 m delay line added to the Sequoia autocorrelator. One SA (always present) is positioned between the booster amplifier (a multipass amplifier located after the oscillator) and the stretcher, to seed the regenerative amplifier with a clean and energetic pulse. Two extra saturable absorbers (SA, RG-850 long pass filter) are inserted in the laser chain between the different preamplifier stages located before the compressor in order to decrease the ASE level and obtain different contrast ratio values for the present study (CR = I_peak_/I_ASE_). Four experimental configurations can be achieved by inserting or removing these SA:

- Two extra SA in the laser chain corresponding to the black curve of laser intensity time evolution in Fig. 6, CR = 3.3 × 10^10^.
- Only one extra SA in the laser chain, which increases the ASE level (red curve in Fig. 6) and so decreases the CR down to 4.2 × 10^9^.
- Zero SA between the preamplifiers. The ASE is again increased (blue curve in Fig. 6). In this case, CR = 1.4 × 10^9^.
- The same configuration as for CR = 1.4 × 10^9^ (only the SA located in the Booster amplifier is present), but the pump energy in the Booster amplifier is reduced. The regenerative amplifier is thus seeded by a train of pulses with moderate energy and the pumping level of the regenerative amplifier is increased to correctly saturate the amplifier. The ASE level is therefore increased, giving CR = 6.7 × 10^7^ (green curve in Fig. 6).

### Characteristics of the laser focal spot

**Figure 7:**
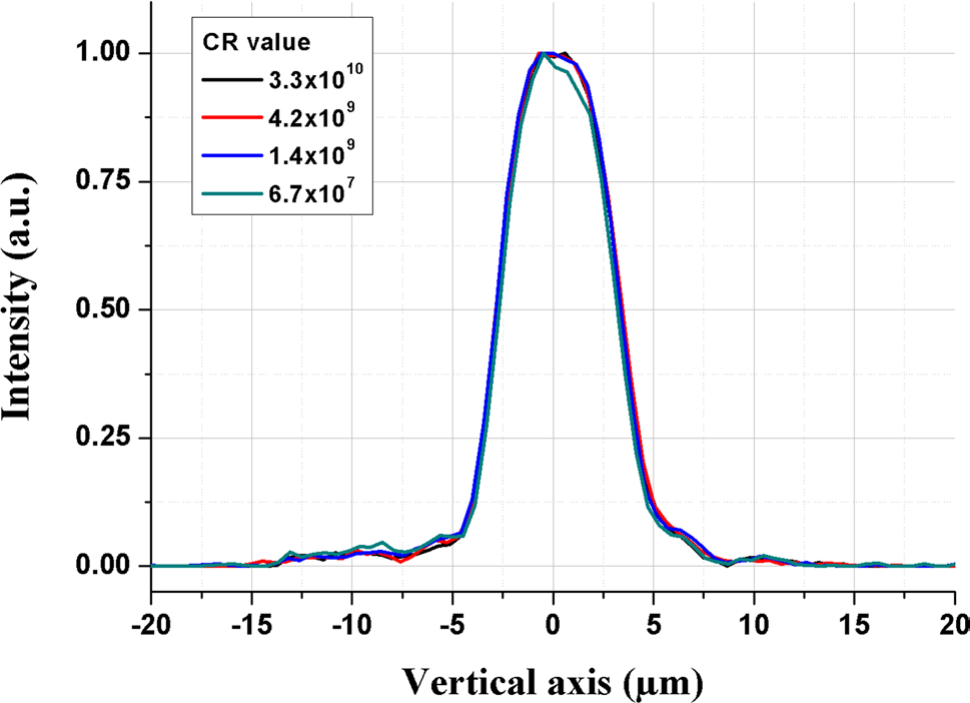
Spatial vertical profile of the laser focal spot for the four temporal contrast values studied. Profiles are CR independent.

The laser focal spot is imaged by a relay imaging system coupled to a beam analyzer with a magnification of 8.4. The detector is a 14 bits CCD device from *Gentec-EO* with 1024 × 1024 pixels of 4.65 × 4.65 µm^2^ surface. Two parallel planar optical uncoated substrates at 45° incident angle are placed after the beam focus to attenuate the reflected beam for spatial characterization, even at full laser energy. A combination of five neutral density (ND) filters *NE10A-B* from *Thorlabs* placed in front of the CCD detector allows to precisely characterize the focal spot up to the maximum pulse energy of 254 mJ. More details about the beam imagery system can be found elsewhere^57^. We also thoroughly characterized the focal spot profile for the different contrast configurations used in this study. These measurements are presented in Fig. 7, here for the vertical profile and for I ~ 1.0 × 10^17^ W/cm^2^.

As shown in Fig. 7, the spatial profile is independent of the temporal contrast configuration. To precisely characterize the laser focal spot wings, some ND filters are removed (up to 3), in order to increase the detection dynamic. The maximal pixel intensity is then saturated revealing lower intensity levels coming from the wings, as we can see in Fig. 3. The profile obtained in this figure is obtained by considering the respective attenuation coefficients of the calibrated neutral density filters that were removed.

### Electron density gradient simulations

**Figure 8:**
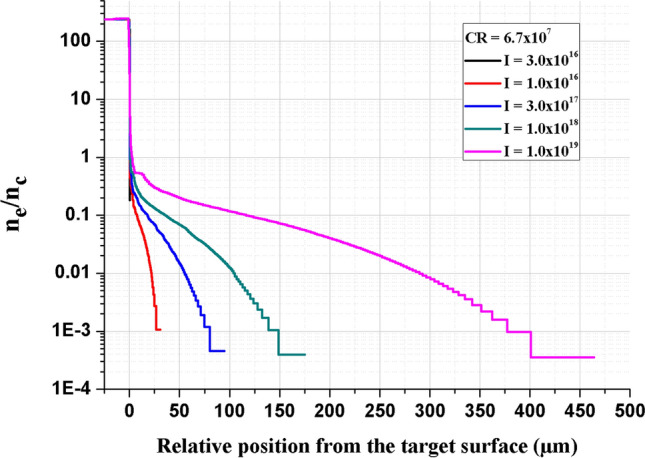
Normalized electron density n_e_/n_c_ as a function of the position from the target surface for CR = 6.7 $\times$ 10^7^ and for the five laser intensities simulated.

The ESTHER code is used to simulate the electron density gradients obtained after the interaction of the ASE and pulse pedestal with the molybdenum target^38^. The experimentally characterized ASE distribution was used in the numerical simulation up to −1.2 ps before the main pulse, which corresponds to the time before the rising edge of the main pulse. Note that the rising time is the same for the four CR. Optical indices for molybdenum are given by Palik in the solid state^58^ and by Faussurier et al*.* in the plasma state^59^. The equations of state (EOS) used come from SESAME, a database developed by the Los Alamos National Laboratory^60^. We also confronted these EOS with those from work of Bushman, Lomonosov and Fortov (BLF)^61^. No significant differences were observed between them concerning the generation of pre-plasma on a molybdenum target. We simulated electron density gradients for the four CR studied in the present paper, and for five laser intensities (3.0 × 10^16^–1.0 × 10^17^–3.0 × 10^17^–1.0 × 10^18^–1.0 × 10^19^ W/cm^2^). As an example, Fig. 8 shows the raw electron density extracted from ESTHER, normalized by the critical density n_c_ for CR = 6.7 × 10^7^. The pre-plasma length L was determined when n_e_/n_c_ = 0.5γ/e, with γ = $\sqrt{1+{\mathrm{a}}_{0}^{2}}$. The dimensionless intensity parameter a_0_ is defined as a_0_ = $\frac{{\mathrm{eE}}_{\rm{L}}}{\mathrm{m}{\upomega }_{\mathrm{L}}\mathrm{c}}$ with e and m respectively the electric charge and the electron mass, c the speed of light, ω_L_ the laser frequency and E_L_ the laser electric field. Note that a_0_ > 1 represents the relativistic laser intensity regime.

### Estimation of the laser intensity I_0_ and of the steepening of the normalized electron density gradient L/λ

The laser intensity I_0,_ for which P_L_ = P_th_, can be analytically estimated. The plasma pressure is defined as P_th_ = $\uprho \frac{{\mathrm{v}}_{\mathrm{s}}^2}{2}$ where v_s_ is the ionic sound speed. The latter is taken as v_s_ = 1.37 × 10^6^Z^1/8^ϒ^-9/16^(Iλτ^1/2^)^1/4^^62^ with Z the target atomic number, τ the laser pulse duration and ϒ = $\frac{Z}{2{[{Z}^{*2}\left({Z}^{*}+1\right)]}^{1/3}}$ where Z* is the effective charge that is determined using the code FLYCHK^63^. For our experimental conditions (Z = 42, λ = 0.8 µm, τ ~ 25 fs and Z* = 6.5), v_s_ = 9.45 × 10^3^ ${\mathrm{I}}_{\rm{ASE}}^{1/4}$ with I_ASE_ the intensity of the ASE. Thus, I_0_ = $\frac{{\rho {\rm c}}}{2{{\rm cos}\theta }}$(9.45 × 10^3^)^2^ ${\mathrm{I}}_{\rm{ASE}}^{1/2}$ with c the speed of light, $\uptheta =45{ \%}$, and ρ = m_e_γn_c_cos^2^θ the mass density, with m_e_ the electron mass, n_c_ the critical electron density, and γ the Lorentz factor.

When the laser intensity I exceeds I_0_, the radiative pressure pushes the pre-plasma back to the target. It is important to evaluate this phenomenon at a crucial time of the interaction corresponding just before the arrival of the main pulse and of the deposition of the laser incident energy into the target. Arbitrarily, we take the time t_0_ equal to ~500 fs before the time corresponding to the maximum peak intensity (see “Methods”, Fig. 6). Indeed, in the temporal interval between t_0_ and the peak of the main pulse, plasma expansion into vacuum related to the rising edge of the main pulse is very limited. For operating conditions corresponding to ours, an ion sound speed of v_s_ = 37 nm/ps was experimentally determined for a pre-pulse developing in the 0–4 ps temporal range before the peak of the main pulse^64^, yielding an estimate of ΔL/λ ≅ 0.02 during 500 fs. This justifies neglecting the influence of any further pre-plasma developing in that temporal range. Note that other choice of t_0_ is not critical and would yield to very similar results and conclusions. Then, an intensity averaged between I_0_ and up to 0.01I_max_ on the rising edge of the main pulse is taken to estimate the squashing of the pre-plasma induced by the laser pulse at a given laser peak intensity I. This choice results in a compromise between a minimal effect related to the application of the laser optical pressure P_L_ at I_0_ and an intensity corresponding to a time ~ 500 fs (or slightly shorter) before the maximum peak intensity (see “Methods”, Fig. 6). We can thus estimate how far the laser pressure crushes the density gradient just before the main pulse thanks to the relation d = v_L_ × t, with v_L_ the velocity associated to the laser pressure and t the rising edge time of the main pulse taken from I_0_ and up to 0.01I_max_. Then, this displacement d is subtracted to the simulated values of L presented in Fig. 2, such as L_d_ = L − d.

### Calculation of the total penetration depth R_total_

${\mathrm{R}}_{\rm{total}}= \frac{{\mathrm{R}}_{\rm{collisional}}{\mathrm{R}}_{\rm{collective}}}{{\mathrm{R}}_{\rm{collisional}}+{\mathrm{R}}_{\rm{collective}}}$ with R_collisonal_= $0.4\times {\mathrm{E}}^{1.265-0.0954\mathrm{ln}(\mathrm{E})}/\uprho$^47^ and R_collective_ = A(T_h_) × z_0_. A(T_h_) is a factor depending to the Maxwell-Jüttner distribution and z_0_ = 3.0 × 10^16^ $\frac{\upsigma {\mathrm{T}}_{\rm{h}}^{2}}{{\upeta }_{\rm{L}\to \mathrm{e}}{\mathrm{I}}_{18}}$, with ${\upeta }_{\rm{L}\to \mathrm{e}}\approx$ 3.8 × 10^–2^ ${\mathrm{I}}_{18}^{3/4}$ the laser to electron conversion efficiency^16^ and σ = 9.7 × 10^3^ $\frac{{\mathrm{T}}_{\rm{e}}^{3/2}}{{\mathrm{Z}}^{*}\mathrm{ln}(\Lambda )}$ the Spitzer conductivity with ln(Λ) the coulomb logarithm, depending on T_e_. T_h_ is determined according to T_h_ ≈ 7 × (I_16_[W/cm^2^]${\uplambda }_{\upmu {\rm{m}}}^{2}$)^1/3^ keV since vacuum heating is the dominant absorption mechanism^42^. The temperature of the plasma layer is calculated using ${\mathrm{T}}_{\rm{e}}= 0.53{\times \mathrm{Z}}^{*1/6}{\mathrm{I}}_{18}^{1/3}{\uplambda }^{-1/6}{\uptau }^{1/6}\mathrm{ keV}$^65^.

## Data Availability

The datasets presented in this current study are available from the corresponding author on reasonable request.
